# Hyaluronate supports hESC‐cardiomyocyte cell therapy for cardiac regeneration after acute myocardial infarction

**DOI:** 10.1111/cpr.12942

**Published:** 2020-10-27

**Authors:** Yuanqing Tan, Lei Wang, Gang Chen, Wenjing Liu, Zhongwen Li, Yukai Wang, Liu Wang, Wei Li, Jun Wu, Jie Hao

**Affiliations:** ^1^ National Stem Cell Resource Center Institute of Zoology Chinese Academy of Sciences Beijing China; ^2^ Institute for Stem Cell and Regeneration Chinese Academy of Sciences Beijing China; ^3^ State Key Laboratory of Stem Cell and Reproductive Biology Institute of Zoology Chinese Academy of Sciences Beijing China; ^4^ University of Chinese Academy of Sciences Beijing China

**Keywords:** clinical‐grade, hESC‐CMs, myocardial infarction, sodium hyaluronic

## Abstract

**Introduction:**

Enormous progress has been made in cardiac regeneration using human embryonic stem cell‐derived cardiomyocyte (hESC‐CM) grafts in pre‐clinical trials. However, the rate of cell survival has remained very low due to anoikis after transplantation into the heart as single cells. Numerous solutions have been proposed to improve cell survival, and one of these strategies is to co‐transplant biocompatible materials or hydrogels with the hESC‐CMs.

**Methods:**

In our study, we screened various combinations of biomaterials that could promote anoikis resistance and improve hESC‐CM survival upon co‐transplantation and promote cardiac functional recovery. We injected different combinations of Matrigel, alginate and hyaluronate with hESC‐CM suspensions into the myocardium of rat models with myocardial infarction (MI).

**Results:**

Our results showed that the group treated with a combination of hyaluronate and hESC‐CMs had the lowest arrhythmia rates when stimulated with programmed electrical stimulation. While all three combinations of hydrogel‐hESC‐CM treatments improved rat cardiac function compared with the saline control group, the combination with hyaluronate most significantly reduced pathological changes from left ventricular remodelling and improved both left ventricular function and left ventricular ejection fraction by 28 days post‐infarction.

**Conclusion:**

Hence, we concluded that hyaluronate‐hESC‐CM is a superior combination therapy for promoting cardiac regeneration after myocardial infarction.

## Graphical abstract

Hyaluronate hydrogels and hESC‐CMs displayed tantalizing prospect in improving cardiac function and delaying left ventricular remodelling after acute MI. And it was the hESC‐CMs that played a major role in left ventricular contraction and improving cardiac function after acute MI, but the hyaluronate hydrogel played an important supporting role.

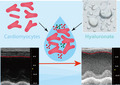

## 1 INTRODUCTION

Due to population ageing and major changes in lifestyles, cardiovascular diseases (CVDs) and especially myocardial infarction (MI) caused by atherosclerosis are now a major cause of morbidity and mortality worldwide.^1^, ^2^, ^3^ It is estimated that there will be more than 23.3 million deaths from CVDs worldwide in 2030.^3^, ^4^ Current treatments for CVD patients include drug regimens, stents, device implantation and heart transplantation. Heart transplantation is the most complete and permanent solution, but it is severely hampered by the lack of organs for donation and other legal‐ethical issues, making it untenable as a solution for the rapidly increasing numbers of CVD patients.^5^ On the other hand, the aforementioned classical treatments are unable to restore damaged cardiovascular tissue and can only delay the progression of CVDs.^3^ Partial regeneration of damaged hearts is an alternative strategy that could avoid these pitfalls and revolutionize CVD therapy. In recent years, tremendous progress has already been made in both pre‐clinical and clinical research on the therapeutic potential of stem cells with respect to cardiac regeneration.^5^, ^6^, ^7^, ^8^, ^9^, ^10^, ^11^, ^12^

However, there are still deep challenges for cell transplantation in cardiac regeneration therapies. One key obstacle is that only a small fraction of the engrafted cells retained at the injection site. For example, only <7% of bone marrow mesenchymal stem cells were detected upon injection into the coronary artery of the patients and only 2% of the stem cells remained 3‐4 days after engraftment.^13^ Typically, there are two ways stem cells can be delivered to the myocardium: intracoronary (IC) infusion and intramyocardial (IM) delivery.^14^ However, the cellular survival rates have remained very low regardless of the delivery route. Only 30%‐40% of the stem cells could be detected at the early stage. Subsequently, the percentage of surviving cells steadily decline, reaching 1% to 15% by 4‐12 weeks.^13^, ^15^, ^16^ Such low survival rates could be caused by many factors, including cell death, ischaemia, immune rejection and 'mechanical' loss during heart beating.^17^, ^18^, ^19^ Teng et al classified the trajectory in post‐implantation cell numbers into three phases, namely phase I, a rapid and massive loss of cells immediately after cell transplantation due to both 'mechanical' loss during heart beating and material loss through the injection orifice; phase II, a period of gradual cell death; and finally phase III, an increase in cell numbers due to cell proliferation.^20^ Many studies have aimed to reduce cell loss in the first two phases,^21^ such as by transplanting cells at the point of cardiac arrest, enclosing the injection orifice with medical biological glue, co‐transplanting cells with biomaterials, treating cells with anti‐apoptosis factors or co‐transplanting cells with factors that promote cell proliferation and inhibit apoptosis.^6^, ^19^, ^22^, ^23^, ^24^ These innovative transplantation strategies have greatly increased the rate of cell retention and survival after injection into the myocardium and improved the recovery of cardiac function.

Recently, some reports have found that hydrogels can activate cell signalling to prevent apoptosis and anoikis by providing a scaffold for cell adhesion.^25^ Matrigel is a mixture of biologically derived extracellular matrix (ECM) proteins which improves cell retention and survival in the infarction area after co‐transplantation with human embryonic stem cell‐derived cardiomyocytes (hESC‐CMs).^6^ Alginate is a natural polysaccharide extracted from algae which forms a matrix after cross‐linking and has been reported to prevent heart deterioration when injected into the infarction area of rat MI models.^26^ Hyaluronate is another natural linear polysaccharide with disaccharide repeats of D‐glucuronic acid and N‐acetyl‐D‐glucosamine, that forms the main component of mammalian ECM. Some studies have shown that hyaluronate could inhibit apoptosis, improve cell survival in the infarction area, promote vascular regeneration and promote recovery of cardiac function when co‐transplanted with cells.^27^, ^28^, ^29^ However, other reports have suggested that inflammation was aggravated after injection of hydrogel into the myocardium.^30^ As a result, the best biomaterial for co‐transplantation with hESC‐CMs for promoting cardiac regeneration had remained unclear.

To screen for the best biomaterial, we cross‐linked three different biomaterials (Matrigel, Alginate and Hyaluronate) to form hydrogels^7^, ^28^, ^31^ and then co‐transplanted the hydrogels with hESC‐CMs into the myocardium of rat MI models. Clinical‐grade functional hESC‐CMs were derived using the VN differentiation system.^12^ Subsequently, cardiac function was evaluated by ultrasound echocardiography, as well as electrocardiography in MI rats with programmed electrical stimulation 4 weeks after transplantation. Our results showed that hyaluronate‐hESC‐CMs provided the best functional outcomes in cardiac regeneration after acute MI in rat models.

## 2 MATERIAL AND METHOD

### 2.1 Ethical Statement

All procedures of this study were completed under the guidelines of the Institute of Zoology, Chinese Academy of Sciences and were approved by the institutional animal care and use committee of the Institute of Zoology, Chinese Academy of Sciences.

### 2.2 Cell culture and differentiation

Our clinical‐grade hESC line (Q‐CTS‐hESC‐2) was maintained in commercially available E8 media on Vitronectin‐NC‐coated plates (1 μg/cm^2^).^32^ Cells were passaged every 5 or 6 days using dispase (1 mg/mL). The cultures were maintained with 3 mL medium per 9.6 cm^2^ of surface area. All cultures were maintained at 37°C, 5% CO_2_ and atmospheric O_2_ in a humidified incubator (Thermo). Cardiac differentiation was performed according to methods previously reported in our laboratory.^12^ Briefly, hESCs were digested into single cells using Accutase (Life Technologies) and reseeded at 10^5^ cells/cm^2^ density on Vitronectin‐NC‐coated plates. The cells were induced to differentiate with VN differentiation medium when they reached 90% confluence after 2‐3 days of culture in E8 medium. In the first 24 hours, the VN medium was supplemented with 4 μM CHIR99021 (Stemgent), which induced hESC differentiation into mesoderm. Two days after, the medium was replaced with VN medium supplemented with 5 μM IWR1 (Sigma‐Aldrich). The medium was changed on day 5, and the IWR1 treatment was maintained for another 3 days. Then, the medium was refreshed every other day with VN medium supplemented with 4 μg/mL insulin. Contractile activity was observed from day 8.

### 2.3 Preparation of cross‐linked biomaterial hydrogels

In our study, we selected sodium alginate and sodium hyaluronate as cross‐linked hydrogels for delivering hESC‐CMs to the myocardium of rat MI models. The cross‐linking was performed as previously reported.^26^, ^31^ Briefly, we prepared 2% sodium alginate solution, 0.6% CaCl_2_ and 2% sodium hyaluronate solution, respectively, and stored them at 4°C. Alginate solution was cross‐linked with CaCl_2_ in a 1:1 volume ratio before co‐injecting with hESC‐CMs.

### 2.4 Echocardiography

Echocardiography data were collected on days −10, −2 and 28 of cell transplantation. Animals were lightly anaesthetized with 5% chloral hydrate each time, and the left ventricular function was measured by paediatric probe (VEVO, 2000) with a 25‐MHz paediatric transducer. Left ventricular fractional shortening (LVFS) was calculated automatically using a software. All measurements were performed by an ultrasound doctor. All operators who performed echocardiographic scans and analyses were blinded to the experimental design.

### 2.5 Animals and surgical procedures

130 male Sprague Dawley rats at the age of 8 weeks were selected in our study. Surgery was performed under general anaesthesia with 5% chloral hydrate. Before surgery, rats were preliminarily assessed using the electrocardiogram from limb leads. The trachea was then exposed for the insertion of trachea cannula if the rat had a normal electrocardiogram. Rats were supported by mechanical ventilation at the set breathing rate of 80 per minute with 1:1 of inspiration and expiration. After opening the chest, the left coronary artery could be seen with the naked eye and the anterior descending branch was ligated with 7.0 suture to induce and model acute MI.^33^ At the end of surgery, the thoracic fluid was absorbed with sterile gauze before closing the sternum and sterilizing the wound site with 75% alcohol. From day −2 to the endpoint of day 28 of cell transplantation, animals were treated with cyclosporine A to suppress the immune response. Rates were injected with 15 mg/kg (i.p.) dose per day in the first week and reduced to 10 mg/kg per day via oral administration thereafter.

### 2.6 Cell transplantation

Q‐CTS‐hESC‐2‐CMs were purified at day 12/13 using the method of discontinuous Percoll gradient as previous reports^34^ and reseeded on Vitronectin‐NC‐coated plates. From 17 to 19 days of Q‐CTS‐hESC‐2‐CMs differentiation, cells were treated as previously reported.^6^ Briefly, one day before transplantation, cells were cultured in medium supplemented with 100 ng/mL IGF1 (PeproTech) and 0.2 mM cyclosporine A (Wako), then heat‐shocked for 30 min at 43°C. The following day, Q‐CTS‐hESC‐2‐CMs were digested into single cells, washed and suspended in 50 μL volume (per animal) of modified medium consisting of either Matrigel, cross‐linked sodium alginate or sodium hyaluronate (50% v/v), and supplemented with 50 nM BCL‐xl BH4 (cell‐permeant TAT peptide, Calbiochem), 200 nM cyclosporine A (Wako), 100 ng/mL IGF1 (PeproTech) and 50 mM pinacidil (Sigma‐Aldrich).

Eight days after surgery to induce acute MI, the rat MI models underwent a repeat thoracotomy, and 2 × 10^6^ cells were injected via five separate injections into the infarcted border and central zone of the free left ventricular myocardium using an insulin syringe with 29‐gauge needle. All groups except for the saline control group were supplemented with pro‐survival cocktails, and the cell therapy groups were mixed with Matrigel, sodium alginate gels and hyaluronate gels, respectively. The surgeon was blinded to the details of each group.

### 2.7 Programmed electrical stimulation

Four weeks after transplantation, the surviving rats were stimulated with programmed electrical stimulation (PES) to detect the stability of cardiac electrophysiology, using methods as previously reported.^5^ In brief, each animal was anaesthetized with 5% chloral hydrate, mechanically ventilated and outfitted for standard limb leads ECG recordings (ADInstruments). Bipolar electrode needles contacted with the cardiac apex and left free wall of left ventricles after thoracotomy. Using standard clinical PES protocols, the pulse output was set at twice the capture threshold, containing a train of eight beats followed by a single extra stimulus for determination of the ventricular effective refractory period (VERP). After that, the heart was challenged three times with a train of eight beats followed by a single extra stimulus (with the S1‐S2 interval set at VERP + 10 ms). If necessary, this procedure was repeated to apply three challenges with double or triple extra stimuli.

After PES, animals were sacrificed and injected with 10% potassium chloride into the ventricles, perfused with saline, followed by tissue fixation using formaldehyde. The infusion needle was inserted at the site of left ventricular apex and the auricula dextra was cut.

### 2.8 Histology and immunocytochemistry

At the day 28 endpoint, all hearts were perfused, the right ventricles and atria were removed and sectioned into five rings from base to apex. The marked ring that had been transplanted with Q‐CTS‐hESC‐2‐CMs was selected, fixed and paraffin‐embedded for histology. The ring was sectioned into 8 μm slices and then prepared for immunohistochemistry. We used primary antibodies directed against cTNT (Abcam) and ZNF397 (Rabbit polyclonal, Sigma‐Aldrich) to identify engrafted Q‐CTS‐hESC‐2‐CMs. Secondary antibodies were diluted with 1% BSA and incubated for 1 hour, nuclei were stained with Hoechst 33342 (10 μg/mL) for 10 minutes and washed, and the slices were covered with coverslips and imaged with an LSM510Meta Confocal Microscope (Zeiss).

### 2.9 Statistics

In our study, one‐way ANOVA of Prism 5.0 was used to analyse the differences between groups with *P* = .05 for significance. All investigators were blinded to the types of data. Values are shown as mean ± SEM, unless stated otherwise.

## 3 RESULTS

### 3.1 Hydrogel‐cardiomyocyte transplantation into rat models of acute myocardial infarction

**FIGURE 1:**
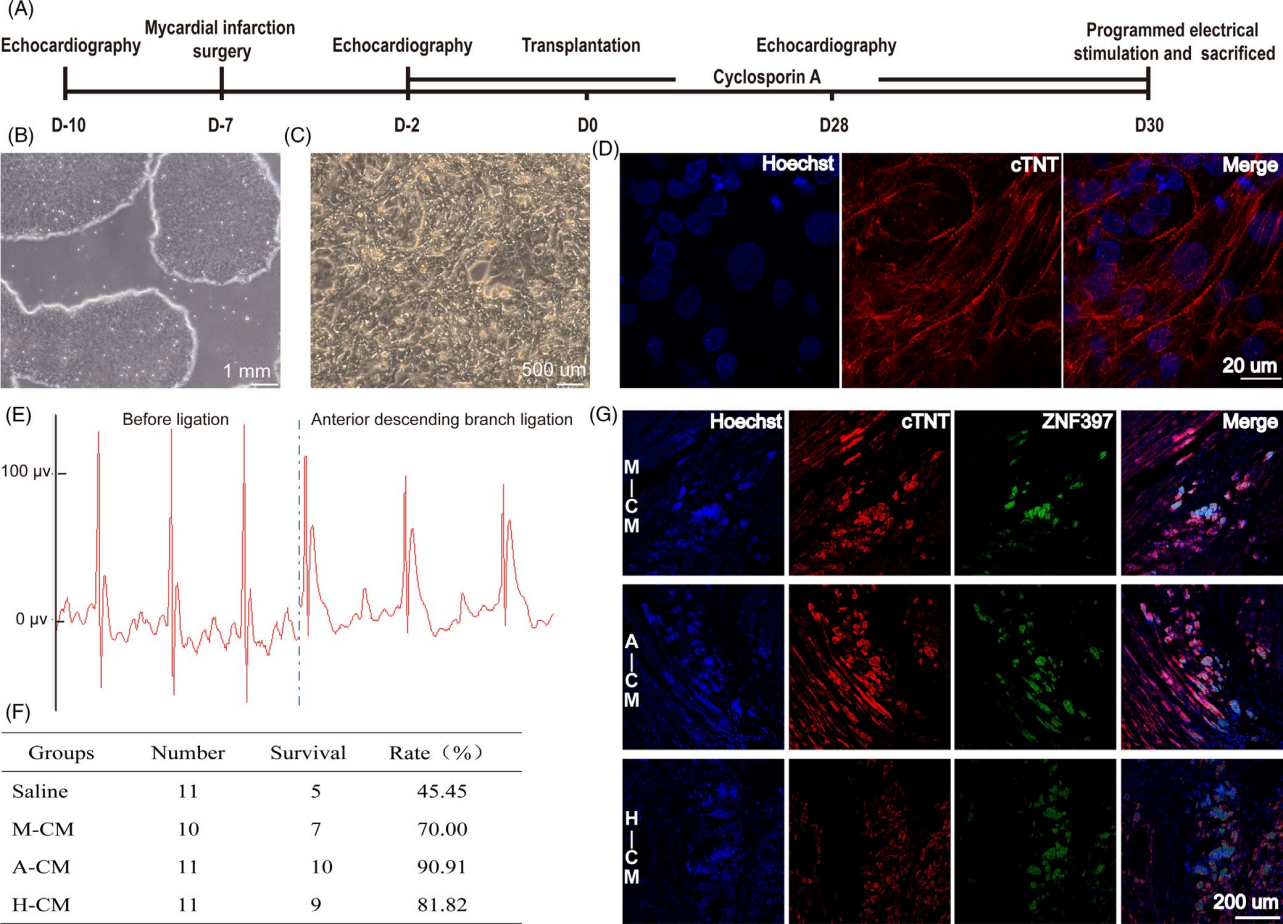
hESC‐CMs transplantation and survival in rat myocardium after co‐transplantation with biomaterial hydrogel. (A) Schematic of study protocol. (B) Representative phase contrast image of human embryonic stem cells (hESCs) before CM differentiation. (C) Representative phase contrast image of hESC‐CMs after differentiation. (D) Representative immunofluorescence image of cTNT expression in hESC‐CMs before transplantation. (E) Electrocardiography after ligation of the anterior descending branch to induce acute MI. (F) Survival rates of rats after surgery and cell transplantation. (G) Double immunofluorescence staining for human‐specific antibody (ZNF397, green) and cTNT (red) of hESC‐CM grafts 4 wk after injection. A, Alginate; CM, hESC‐derived cardiomyocytes differentiated using the VN differentiation system; cTNT, cardiac troponin T; H, Hyaluronate; M, Matrigel

Clinical‐grade human embryonic stem cells, Q‐CTS‐hESC‐2, were derived under xeno‐free conditions in our laboratory, and used for CM differentiation (Figure 1A‐D). We prepared 130 male Sprague Dawley rats, of which 5 rats were excluded as their left ventricular ejection fraction were already under 55% even before disease modelling. 7 rats showed abnormal electrocardiograms when placed under general anaesthesia. The remaining 118 rats underwent thoracotomy. We ligated the anterior descending branch with 7/0 wires to induce acute myocardial infarction (MI). Electrocardiography with limb leads demonstrated abnormal ST segment and T waves, indicating that the modelling of acute MI was successful (Figure 1E).

Five days after induction of acute MI, the surviving rats were narcotized and their left ventricular ejection fraction (EF) data were evaluated. Rats that met the criterion of 25% ≤ EF ≤ 45% were randomized into four groups: saline control group, matrigel + hESC‐cardiomyocyte (M‐CM) group, alginate + hESC‐cardiomyocyte (A‐CM) group and hyaluronate + hESC‐cardiomyocyte (H‐CM) group. Cell transplantation was then performed over the next two days. Interestingly, rats that received hydrogel‐CM injections had higher survival rates after acute MI (Figure 1F). After 28 days, we extracted the cardiac tissue and detected surviving human cardiomyocyte grafts in the infarcted rat myocardium of all three groups implanted with both hydrogels and hESC‐CMs (Figure 1G).

### 3.2 Acute MI rat left ventricular ejection fraction after co‐transplantation with hydrogels

**FIGURE 2:**
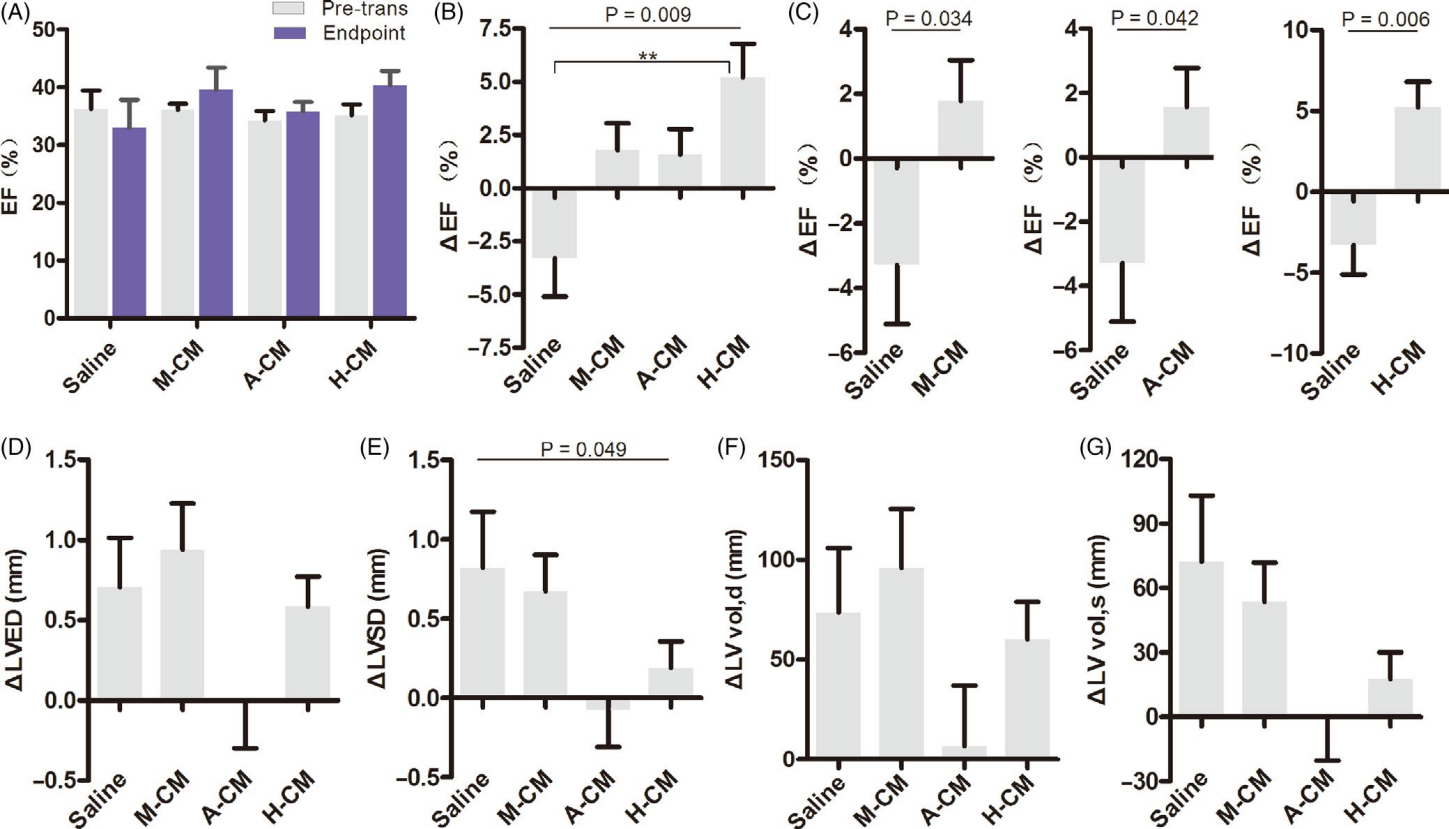
Echocardiography of ventricular function after acute MI and hydrogel‐CM transplantation. (A) Co‐transplantation of hydrogels and hESC‐CMs can promote left ventricular recovery in average EF at the 28‐day endpoint, relative to the pre‐transplantation state. (B) Co‐transplantation of hydrogels and hESC‐CMs can promote left ventricular recovery in ΔEF (%change in EF post‐transplantation for each rat). **P < .01, one‐way ANOVA. (C) Co‐transplantation of M‐CM, A‐CM and H‐CM can promote left ventricular recovery in ΔEF. *P*‐value, Student *t* test. (D‐G) Comparison of absolute changes in LVED, LVSD, LV vol,d, LV vol,s among the four treatment groups. EF, left ventricular ejection fraction; LV vol,d, Left ventricular end diastolic volume; LV vol,s, Left ventricular end systolic volume; LVED, left ventricular end diastolic diameter; LVSD, Left ventricular end systolic diameter

Four weeks after cell transplantation, the surviving rats’ cardiac functions were measured with ultrasound echocardiography and myocardial electrophysiological stability was measured using programmed electrical stimulation. In a previous study, we reported that co‐transplantation of cells with Matrigel significantly improved cardiac function in rats, compared to saline and Matrigel alone.^12^ In this study, we obtained similar results. The average EF decreased from 36.23 ± 7.14% to 32.94 ± 10.96% in the saline group after acute MI, whereas the average EF of three hydrogel‐CM co‐transplantation groups increased from ~34%‐36% to 39.55 ± 12.12%, 35.82 ± 5.18% and 40.33 ± 7.41% for M‐CM, A‐CM and H‐CM, respectively (Figure 2A). While the ΔEF (percentage change in EF post‐transplantation for each rat) in groups receiving hydrogel‐CM all showed recovery compared to the saline group, the strongest and most statistically significant recovery was observed for the H‐CM group of rats (Figure 2B‐C).

These results demonstrate that co‐transplantation of hESC‐CMs with biocompatible hydrogels into the myocardium can prevent left ventricular function from further deterioration after acute MI in vivo, and hyaluronate had the best effect in improving cardiac function among the three biomaterials.

### 3.3 Left ventricular remodelling in acute MI rats after co‐transplantation with hydrogels

Left ventricular remodelling tends to occur after myocardial infarction. Maladaptive ventricular cardiomyocyte hypertrophy and scar tissue formation in the infarcted region causes expansion in the left ventricles, eventually resulting in chronic heart failure. Here, we measured the relative parameters of left ventricular remodelling in acute MI rats after injecting the mixtures of hydrogel‐hESC‐CMs (Table S1)

. The left ventricular end systolic/diastolic diameters and volumes all increased in the saline group, suggesting that our surgical modelling of acute MI successfully led to left ventricular remodelling (Figure 2D‐G). Co‐transplantation with Matrigel (M‐CM) failed to prevent left ventricular remodelling (Figure 2D‐G). On the other hand, co‐transplantation with alginate (A‐CM) effectively stopped left ventricular remodelling, without any increase in the left ventricular end systolic/diastolic diameters and volumes (Figure 2D‐G). Co‐transplantation with hyaluronate (H‐CM) slightly ameliorated the increase in diastolic diameter and volume, and significantly decreased the systolic diameter and volume (Figure 2D‐G). These results illustrated that alginate hydrogel c‐transplantations can effectively prevent left ventricular remodelling after acute MI, whereas hyaluronate hydrogel can delay the process.

### 3.4 Left ventricular contraction in acute MI rats after co‐transplantation with hydrogels

**FIGURE 3:**
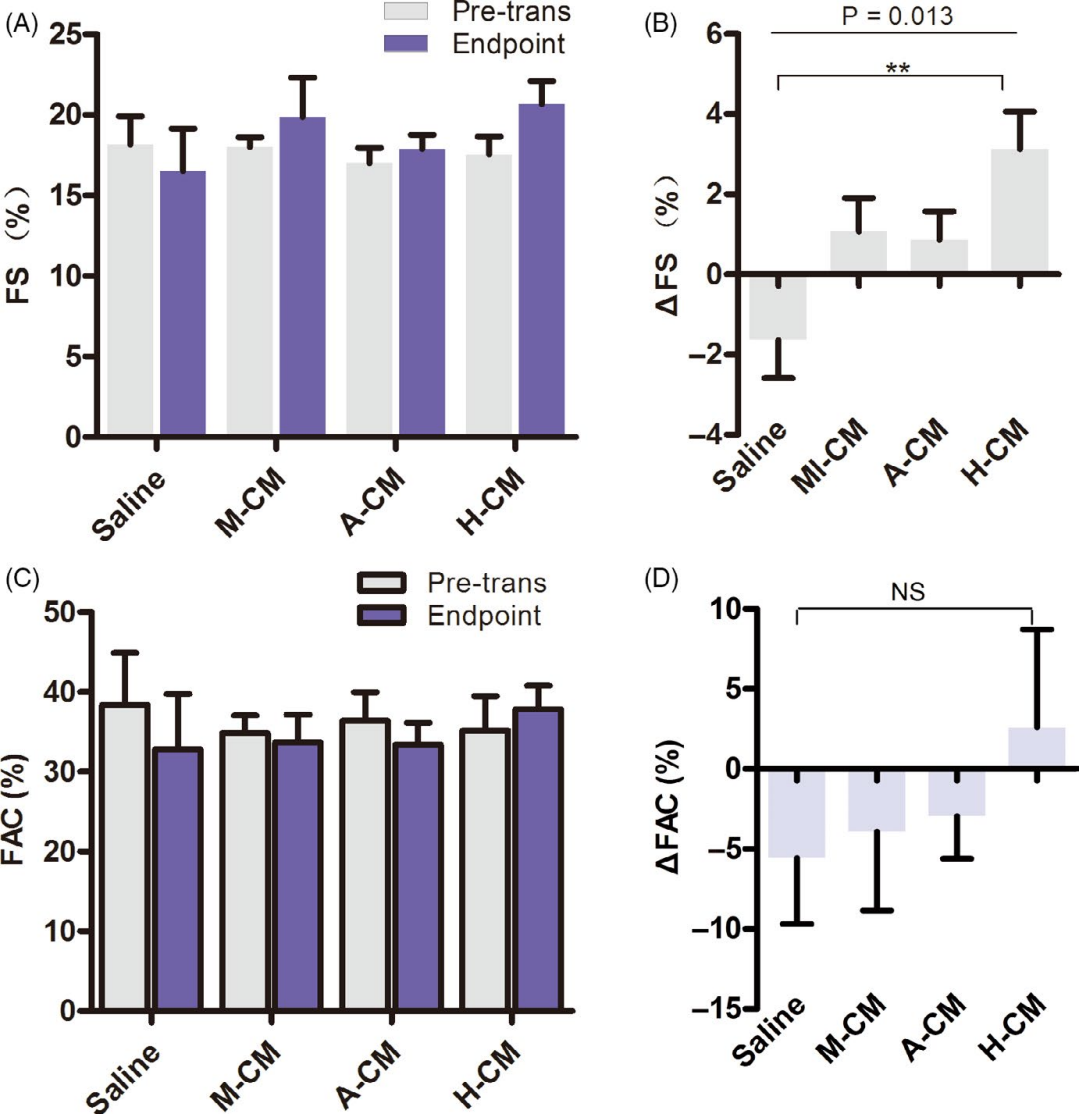
Echocardiography of left ventricle systolic function after acute MI and hydrogel‐CM transplantation. (A) Co‐transplantation of hydrogels and CMs can promote recovery of left ventricular average FS in vivo at the 28‐day endpoint, relative to the pre‐transplantation state. (B) Co‐transplantation of hydrogels and CMs can promote recovery of left ventricular ΔFS (%change in FS for each rat) in vivo. (C) Co‐transplantation of hyaluronate and CMs can promote recovery of left ventricular FAC in vivo at the 28‐day endpoint, relative to the pre‐transplantation state. (D) Co‐transplantation of hydrogels and CMs can promote recovery of left ventricular ΔFAC in vivo. FAC, ventricular fractional area change; FS, left ventricular fractional shortening

Based on the above results, we further analysed left ventricular fractional shortening (FS), that is the percentage of size differences of the left ventricle as an indicator of left ventricle contractile function during systole, after acute MI. The FS was significantly decreased in the saline group, from 18.15 ± 3.93% at pre‐transplantation to 16.51 ± 5.9% at the end of the experiment. In contrast, the FS showed varying degrees of improvement in the other three groups 4 weeks after transplantation. The H‐CM group showed the greatest increase, from 17.54 ± 3.28% to 20.66 ± 4.30%, while the other two groups increased from 18.02 ± 1.87% to 19.87 ± 7.74% and 17.02 ± 2.88% to 17.88 ± 2.83% in M‐CM and A‐CM, respectively (Figure 3A). On a per rat basis, the H‐CM group also displayed the largest increase in ΔFS among the three biomaterial co‐transplantation groups (Figure 3B, Figure S1), whereas no differences were observed when the other two groups were compared to the saline group.

Ventricular fractional area change (FAC), assessed by ultrasound echocardiography, is another assessment of cardiac contractile function. The results indicated that FAC decreased in all groups except the H‐CM group at 4 weeks after transplantation (Figure 3C). Although we did not find significant differences when analysing ΔFAC on a per rat basis (Figure 3D), the above FS and FAC data led us to conclude that co‐transplantation of hESC‐CMs with hyaluronate hydrogel into the myocardium of acute MI rat models had a positive effect in improving ventricular contractile function.

### 3.5 Left ventricular function in acute MI rats after injection of hyaluronate hydrogel alone

**FIGURE 4:**
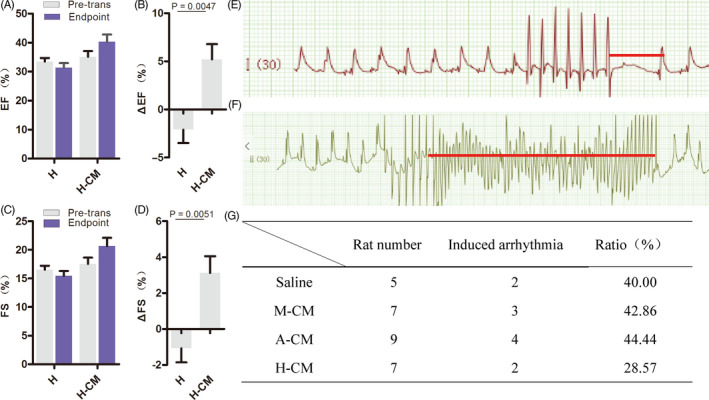
CMs play a major role in the pro‐regenerative effect of H‐CMs in improving cardiac function after acute MI. (A) Only co‐transplantation of hyaluronate and hESC‐CMs can promote left ventricular recovery in average EF at the 28‐day endpoint, relative to the pre‐transplantation state. (B) Only co‐transplantation of hydrogels and hESC‐CMs can promote left ventricular recovery in ΔEF (%change in EF for each rat). (C) Only co‐transplantation of hyaluronate and CMs can promote recovery of left ventricular average FS in vivo at the 28‐day endpoint, relative to the pre‐transplantation state. (D) Only co‐transplantation of hyaluronate and CMs can promote recovery of left ventricular ΔFS (%change in FS for each rat) in vivo. (E) Measurement of the maximum effective refractory period (red line) using the S1S2 simulation model. (F) Sustained ventricular tachycardia was induced after programmed electrical stimulations (PES; red line). (G) The numbers and ratios of induced arrhythmias in all 4 groups of treated rats. H, sodium hyaluronate

Given the above data, the combination of hyaluronate hydrogels and hESC‐CMs displayed the best results in improving cardiac function and delaying left ventricular remodelling after acute MI. To discern the respective contributions of hyaluronate and cardiomyocytes in cardiac regeneration and improving cardiac function, we designed another experimental group, where we only injected hyaluronate hydrogel alone into the rat myocardium after acute MI. The results showed that both the EF and the FS further decreased when only hyaluronate hydrogel was used (Figure 4A‐D). While the differences in average EF and average FS after transplantation were not significant when compared to H‐CM (Figure 4A,C), the ΔEF and ΔFS were significantly higher in the H‐CM group than in the H group when assessed on a per rat basis (Figure 4B,D). These results indicate that the hESC‐CMs played a major role in cardiac regeneration and improving cardiac function after acute MI and that the hyaluronate hydrogel played a supportive role.

### 3.6 Hyaluronate‐cardiomyocytes protect against arrhythmias after acute MI

Arrhythmia is one of the lethal complications of acute MI because electrical conductance defects around the infarcted zone of the heart can lead to instability of overall cardiac electrophysiology. We induced and detected arrhythmias using programmed electrical stimulation (PES) in all 4 treated groups of acute MI rats (ref; Figure 4E‐F). Induced arrhythmia was detected in all 4 groups, but the H‐CM group had the lowest ratio of induced arrhythmias (Figure 4G).

## 4 DISCUSSION

The overall objective of this study is to screen for a suitable biomaterial that can be co‐transplanted with hESC‐CMs into animal models of acute MI in vivo and provide a reference for future clinical research. Cell therapy for CVD faces many challenges, such as mechanical loss from heart beating and cell death from stem cell anoikis, inflammation and immune rejection. These negative factors suppress the curative effects of cell therapy due to the low survival rate of cells in the damaged zone after transplantation. Recently, scientists have found that combinations of cells and biomaterials can improve cell survival and increase cell retention by simulating the cellular microenvironment and activating anti‐apoptosis signalling.^23^, ^24^, ^29^, ^35^ The biomaterial previously used to deliver hESC‐CMs in pre‐clinical trials was Matrigel.^5^, ^6^, ^7^ Matrigel is a colloidal biological mixture, which consists of extracellular proteins derive from mouse sarcoma tumour cells.^36^ But it is impractical to use Matrigel for clinical applications because it contains many undefined types of extracellular matrix proteins, oncogenic growth factors and other undefined ingredients.^37^ Therefore, it is important for us to screen for a natural biomaterial to aid the delivery of hESC‐CMs.

Alginate is a natural biological polysaccharide which is stable, soluble, viscous and safe for use as a pharmaceutical excipient. Moreover, as a cross‐linked hydrogel,^38^, ^39^ alginate prevents adverse cardiac remodelling and dysfunction both shortly and long after acute MI in rats.^26^ Hyaluronate‐based gels are also appealing for co‐injection, as this glycosaminoglycan polymer is one of the main components of naturally occurring extracellular matrix within mammalian connective tissues. It has been shown to promote angiogenesis in infarcted hearts, improve cell retention and survival, and left ventricular function.^26^, ^27^, ^28^, ^29^ Based on these findings, we selected alginate and hyaluronate‐based biomaterials and cross‐linked them to form hydrogels.^26^, ^31^ The resultant hydrogels were formulated and co‐injected with hESC‐CMs into the myocardium of rat acute MI models. We found that the combination of alginate and hESC‐CMs effectively prevented left ventricular remodelling. It has been previously reported that injection of alginate hydrogels into the infarcted zone of rat acute MI models^26^ can prevent cardiac deterioration. This was similar to what we observed. However, we found that the ventricular functional recovery was not as pronounced in the alginate co‐transplantation group as other hydrogel co‐transplantation groups. Consistent with our previous study, we found that co‐delivery of hESC‐CMs and Matrigel to the infarcted zone can also improve cardiac functional recovery. However, in this study, we demonstrated that hyaluronate hydrogel was the best among the biomaterials we screened for supporting hESC‐CMs in cardiac regeneration after acute MI. The combination of hESC‐CMs and hyaluronate‐based hydrogel was the best in improving cardiac functional recovery, delaying left ventricular remodelling and preventing arrhythmias in rat acute MI models. While it is clear that hESC‐CMs play the major role whereas hyaluronate plays the supportive role in cardiac regeneration after acute MI, the molecular mechanisms for this supportive function remain unclear. There are some reports suggesting that hyaluronate is one of the main components of the heart ECM, thus mediating cellular adhesion, self‐renewal, differentiation and migration by providing a suitable microenvironment for cardiomyocytes.^40^, ^41^, ^42^ In addition, hyaluronate can also be degraded rapidly in vivo and its degradation products can promote angiogenesis and cardiac regeneration.^40^ In addition, it has been reported that hyaluronate rapidly restores metabolism of stem cells when co‐cultured in vitro.^43^ All of the above hypotheses may be possible mechanisms for the superior performance of the combination of hyaluronate and hESC‐CMs in improving cardiac functional recovery after acute MI in vivo.

Programmed electrical stimulation is an important method to test the stability of cardiac electrophysiology. In our study, induced ventricular tachycardia was detected in all groups. It is known that hESC‐CMs can aggregate and form cell islets upon retention in the infarcted area, thus increasing the risk of arrhythmia.^6^, ^7^ In addition, injected biomaterials may persist for a long time within the myocardium and may induce inflammation in the process.^44^ Previous reports suggest that injection of synthetic hydrogels can worsen inflammation in the injected zone, suggesting that exogeneous hydrogels are not always beneficial for the heart,^30^ and could disturb the electrical coupling between cardiomyocytes and hence induce arrhythmia.^21^ In our study, acute MI rats that were co‐injected with hyaluronate and hESC‐CMs displayed the most stable cardiac electrophysiology and had the lowest rates of induced arrhythmias when stimulated. This could be because hyaluronate degrades rapidly in vivo within 12 hours after injection, and it is completely degraded within 13 days.^45^ Hence, hyaluronate is the least likely biomaterial to cause inflammatory responses within the heart.^45^, ^46^ This could explain the lowest rates of induced arrhythmias in the group co‐injected with hyaluronate and hESC‐CMs.

In conclusion, we discovered that hyaluronate‐based hydrogel is the most suitable biomaterial for delivering and supporting hESC‐CMs in cell therapy for acute MI in vivo. Further work will be needed to explore the mechanism(s) underlying hyaluronate's role in supporting hESC‐CMs during cardiac regeneration and functional recovery after acute MI.

## CONFLICT OF INTEREST

The authors declare that there is no conflict of interest that could be perceived as prejudicing the impartiality of the research reported.

## AUTHOR CONTRIBUTIONS

JH, WL and JW conceived the project. YT, LW, GC and Wen.L. performed the experiment. ZL, YW and Liu.W. analysed data. YT and LW designed the research and wrote the manuscript with help from all of the authors. YT and LW contributed equally to this article. J. H. and JW are the corresponding authors of this article. All authors read and approved the final manuscript.

## Supporting information

Fig S1

Table S1

## Data Availability

The data that support the findings of this study are available from the corresponding author upon reasonable request.
