# Supplementary figures and images for: Hyaluronate supports hESC‐cardiomyocyte cell therapy for cardiac regeneration after acute myocardial infarction
Source: Cell Prolif. 2020 Oct 27;53(12):e12942. doi: 10.1111/cpr.12942 (PMC7705924; doi:10.1111/cpr.12942)

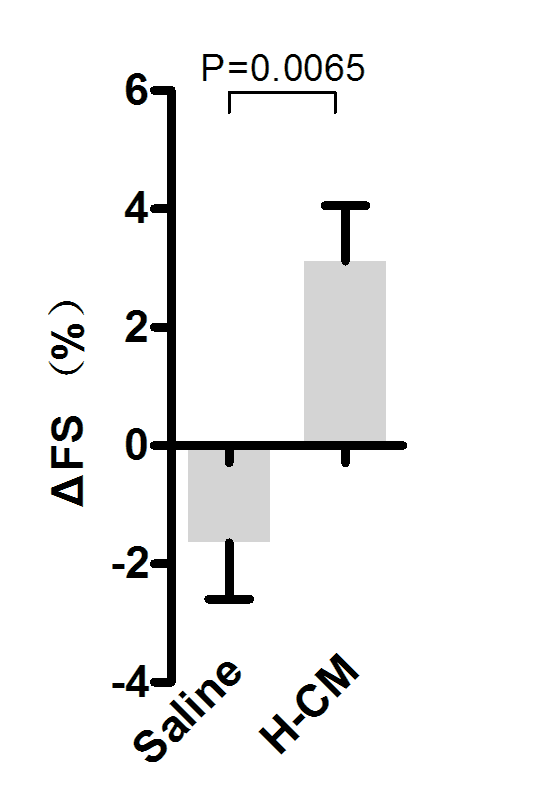

Supplement: Supplementary file 1 — Fig S1 [file CPR-53-e12942-s001.tif]
