# Supplementary material for: Hyaluronate supports hESC‐cardiomyocyte cell therapy for cardiac regeneration after acute myocardial infarction
Source: Cell Prolif. 2020 Oct 27;53(12):e12942. doi: 10.1111/cpr.12942 (PMC7705924; doi:10.1111/cpr.12942)
Supplement: Supplementary file 2 — Table S1 [file CPR-53-e12942-s002.docx]

**SUPPLEMENTARY TABLE 1 Left ventricular diameters and volumes**

|  |  | **LVED (mm)** | **LVSD (mm)** | **LV vol,d (μL)** | **LV vol,s (μL)** |
| --- | --- | --- | --- | --- | --- |
| **Saline** | Pre-trans | 7.70±0.51 | 6.25±0.61 | 318.36±45.04 | 199.93±43.50 |
|  | Endpoint | 8.41±1.14 | 7.07±1.38 | 391.99±110.54 | 272.14±111.13 |
| **M-CM** | Pre-trans | 7.64±0.52 | 6.23±0.42 | 312.85±50.06 | 196.97±31.50 |
|  | Endpoint | 8.58±0.99 | 6.90±0.75 | 408.74±102.13 | 250.62±59.36 |
| **A-CM** | Pre-trans | 7.72±0.36 | 6.42±0.45 | 319.48±32.91 | 211.21±34.04 |
|  | Endpoint | 7.72±1.11 | 6.35±1.0 | 326.13±111.42 | 210.71±82.65 |
| **H-CM** | Pre-trans | 7.85±0.49 | 6.48±0.56 | 331.91±46.59 | 216.6±42.64 |
|  | Endpoint | 8.44±0.83 | 6.67±0.91 | 391.97±84.87 | 234.21±71.08 |

LVED, left ventricular end diastolic diameter; LVSD, Left ventricular end systolic diameter; LV vol,d, Left ventricular end diastolic volume; LV vol,s, Left ventricular end systolic volume.
